# Lecture on Materia Medica

**Published:** 1855-10

**Authors:** H. Bence Jones

**Affiliations:** Physician to St. George’s Hospital


*Lecture on Materia Medica.* Given at the Royal College of Physicians. By H. BENCE JONES, M. D., F. R. S., Physician to St. George's Hospital.

### ON DIGITALIS.

It is my intention to-day to take as my subject the effects of Digitalis, partly on account of the interest which its medicinal action possesses, partly because some accurate experiments have lately been made in Germany and in France on the use of this medicine, which, so far as I know, have not been made known here.

This volume of the *Archives de Physiologie*, edited by M. Bouchardat, containing the memoir of MM. Homolle and Quevenne on Digitaline and Digitalis, might alone have furnished me with the materials for this lecture; I shall, however, bring before you not only the most important facts determined by these investigators; but I shall first relate to you some remarkable results obtained by Dr. Traube of Berlin, respecting the action of digitalis on some animals.

The experiments of Dr. Traube were chiefly made on dogs. The infusion of digitalis which he used was prepared by pouring four ounces of boiling water on two drachms of digitalis leaves; the infusion was carefully filtered, and was raised to the temperature of the body before it was injected into the veins by a syringe which held 130 grains of spring water. Thus each syringe-full of the infusion digitalis would contain the extract from about eight grains of digitalis leaves. The infusion was injected into the jugular vein towards the heart.

A strong dog had, first, a syringe-full of warm salt and water injected into the vein. The pulse was slightly accelerated; then a syringe-full of infusion of digitalis was injected, and in one minute the pulse fell 108 pulsations. In ten minutes, on the pulse rising, two thirds of a syringe-full more were injected, and the pulse fell 28 beats. This was again repeated; after the fifth syringe-full the pulse rose above what it was previous to the commencement of the experiment.

The details of the experiment may be thus given:—

| | A. M. | | |
|---|---|---|---|
| The injecting tube was fixed at | 7.56. | Then pulse, | 128 |
| Warm salt and water injected | 8.16. | " | 128 |
| 1st syringe-full of digitalis | 8.34. | " | 132 |
| " " | 8.35. | " | 24 |
| 2d syringe, two-thirds full | 8.46. | " | 84 |
| " " | 8.48. | " | 56 |
| 3d syringe, one and half full | 8.54. | " | 84 |
| " " | 8.55. | " | 36 |
| 4th syringe-full, - - - | 8.57. | " | 32 |
| 5th syringe-full, - - - | 9.01. | " | |
| " " | 9.04. | " | 160 |
| " " | 9.07. | " | 174 |
| At | 9.17 dog was bled to death. | | |

In this experiment the effect of increasing the quantity of digitalis are very remarkable.

In another experiment, the same fact is thus made evident:

At first the pulse was 108. With two and one-third syringesfull the pulse fell to 33. With two-thirds of a syringe-full more, the pulse rose to 202.

In a third experiment, the pulse before injection was 132: after three-syringes-full, it was 46; after four syringes-full were injected, it was 192; after five syringes-full, it rose to 204.

The fourth experiment gave the same result.

In the fifth, the pulse before injection was 128; after a syringe and a-half was injected, the pulse fell to 72; after nearly four syringes-full, the pulse rose to 100; after five syringes-full, the pulse reached 216. The dog lived eleven hours afterwards. Two other experiments all agreed in showing that the first action of small quantities of digitalis injected into the blood was to reduce the pulsation of the heart; but that, when the quantity of digitalis was increased, the pulsations of the heart became greatly accelerated.

Having determined this action of digitalis, Dr. Traube then endeavored to satisfy himself whether this effect on the pulse depended on the action of the digitalis on the heart directly, or on the heart through the pneumogastric nerves. One series of experiments was made, in which the pneumogastric nerves were divided after the digitalis was injected, and, in another series, the pneumogastrics were divided before the injection of the infusion.

The first of these series consisted of seven experiments. The following may be taken to illustrate the effects:—

When the pulse was reduced by the action of the injection of the infusion, first one, and then the other pneumogastric nerve was divided; and the results are thus stated:—

| | |
|---|---|
| The pulse before the injection of the infusion was | 72 |
| " after " " | 44 to 52 |
| Immediately, on sect. of left vagus, pulse rose to | 92 |
| " " right " " | 204 |

In another experiment:—

| | | | |
|---|---|---|---|
| Before the injection of the infusion, at | 2.8 p. m., | the pulse was | 121 |
| After ⅔ of syringe-full at | 2.11 | " | 88 |
| " " | 2.14 | " | 50 |
| " " | 2.16 | " | 48 |
| " " | 2.17 | " | 48 |
| The right vagus was cut at | 2.19 | " | |
| " " | 2.21 | " | 66 |
| The left vagus was cut at | 2.27 | " | 204 |

The same result was obtained by dividing both pneumogastric nerves at the same time. In all the seven experiments, after the pulse was reduced by the action of digitalis, division of the vagi caused rapid acceleration of the heart's action.

When the pneumogastric nerves were divided, previously to the injection of the infusion of digitalis, the reduction of the pulse was no longer observable.

The following experiments illustrate the results which were then obtained:—

| | P. M. | | |
|---|---|---|---|
| Both pneumogastrics were divided at | 3.56 | Pulse was | 180 |
| Half a syringe-full was injected | 4. 8 | " | 168 |
| Another half " | 4.16 | " | 162 |
| One-third syringe-full " | 4.23 | " | 152 |
| Half a syringe-full " | 4.32 | " | 160 |
| Half a syringe-full " | 4.41 | " | 180 |
| Half a syringe-full " | 4.49 | " | 180 |

Twelve experiments gave the same results. Here is the last.

Before injection, after division of vagi. Pulse was 180.

| | | |
|---|---|---|
| Two syringes-full at | 9.15 A. M. | |
| " | 9.17 " | Pulse was 144 |
| " | 9.19 " | " 126 sys. mur. |
| " | 9.20 very loud systolic murmur. Second sound wanting. | |

After four more syringes-full. Pulse 144.

It follows, from the first series of these experiments, that digitalis, in small quantity, reduces the action of the heart; in large quantity, greatly accelerates it.

It follows, from the second series of these experiments, that when the digitalis has reduced the pulse, division of the vagi will immediately insure acceleration.

It follows from the third series of these experiments, that when the vagi are divided, digitalis is not observed to produce slowness of the pulse.

In the interpretation of these results, two fundamental observations by other physiologists must be here mentioned.

E. Weber has shown that, when the vagi are undivided, a feeble electric current acting on the medulla oblongata or on the vagi, causes a diminution of the contractions of the heart.

Ludwig has proved that, in all mammalia, section of the vagi in the neck is immediately followed by increased frequency of the heart's action.

We must recognize in the heart two systems of nerves:—

1st. Musculo-motor, causing contraction.
2nd. Regulator system.

The ganglia of the heart are the centre of the 1st system, and the medulla oblongata is the centre of the 2nd system. The regulating nerves pass with the vagi. From the experiments of Weber and Ludwig, it follows, 1st. That abnormal gentle stimulus of the regulator-nerves diminishes the frequency of the heart's action, and 2ndly. That the frequency is greatly increased by the removal of the regulating action.

From this it may be concluded, that any substance which, when brought into circulation in small quantity, diminishes the frequency of contraction, but, in large quantity, increases the frequency, acts on the regulator-nerves. Hence digitalis, from Dr. Traube's experiments, 1st. Stimulates the regulator system of nerves; 2ndly. Paralyzes the regulators; and when it stops the action of the heart, then, 3rdly. It paralyzes the musculo-motor system.

In small doses, the digitalis acts as a stimulant; in large doses, it acts as a sedative paralysis and death. Though it is by no means safe to deduce the action of any medicine on man from the effect of the same medicine on animals, as indeed the action, or rather the absence of action, of this very substance (digitalis) on rabbits well proves. Yet the phenomena produced by large doses of digitalis on man so closely resemble those produced on dogs by injection, that it may safely be assumed, that in man digitalis acts on the nerves that regulate the heart's action, first as a stimulant, and in large doses as a sedative. I will pass on, therefore, now to the consideration of digitalis as a medicine. The uncertainty of the action of remedies is often rightly attributed to variations in their composition. In all vegetable medicines, used as they are grown, this is most likely to occur. And no certainty regarding the mode of action of any vegetable medicines can be obtained until the active substances are separated and made the subject of careful experiment. I might take opium and bark to illustrate my remarks. We do not, indeed, as yet perfectly understand the mode of action of morphine and quinine, but by separating these substances, it has become possible for us to determine how opium and bark act on the system; and in the use of these medicines we can get more constant effects by more constantly using exactly the same remedy So also, if we can get the active principle of digitalis, we shall not only advance on the way to the knowledge of the mode of action of digitalis, but we shall get rid of causes interfering in the action of this substance as a remedy; for example, the age of the plant, the year, the cultivation of the plant, the desiccation of the leaves, &c.

M. M. Homolle and Quevenne, to whom we are indebted for this important work on digitalis, state that they have separated fourteen different substances from the leaves of the plant; as the amount of each of these substances present in the plant is never constantly the same, it is evident that much more certainty will be obtained by insulating the one active substance, than by using fourteen substances in various quantities.

The different substances found by the French chemists may be thus enumerated: starch, sugar, pectin, albuminous matter, orange-red crystallizable coloring matter, chlorophyle, volatile oil, tannic acid, digitalic acid, anterrbinic acid, digtalic, and three neutral principles, digitalose, digitalide and digitaline.

This last substance is the most active ingredient of the digitalis. It is prepared pure by extracting the neutral principles with ether and alcohol, of specific gravity 780. This dissolves the digitaline and the digitalose, the etherial solution is then evaporated, the residue treated with alcohol, again evaporated, and treated with weak alcohol, the digitaline remains dissolved. On gentle evaporation, it does not crystallize, but forms a resinous-looking mass of a pale yellow color, unchangable in the air, and very bitter, slightly soluble in water, very so'u le in alcohol; It is a neutral substance. It becomes emerald-green with strong hydrochloric acid. The best test of its quality is its bitterness. This is best determined by taking one centigramme of the powder of digitaline, and dissolving it in two grammes of alcohol, and continuing to dilute this solution with water until the bitterness is found to disappear. From the quantity of water required, the goodness of the digitaline may be estimated; if the digitaline be good, more than three pints of water will be required to be added before the bitterness becomes imperceptible.

The best form for keeping and giving the digitaline as medicine is in granules and not tincture. Thus it keeps best, and is more certain, in composition. It is thus most easily given, as its bitter taste is concealed. Each granule is made to contain one millegramme. This is equal to .015 grain of digitaline. Here are the granules as prepared by M. Homolle, and here are some prepared by Mr. Morson, each granule containing the hundredth part of a grain of digitaline. I have used both these preparations in all kinds of diseases in St George's Hospital, and I can find no difference in their action.

MM. Homolle and Quevenne state that one of them took four of these granules daily for eight days. The average healthy pulse of the person experimented on was 67.5; after taking the granules the pulse fell to 50. The difference is 17.5 beats, which are equal to one quarter the beats of the heart. Two dogs were given from 2 to 11 granules daily. In the first dog the pulse fell from 60 to 51. In the second, from 87 to 70.

The comparison between the digitaline and digitalis is so remarkable that I have copied it in this table.

| Digitaline. | Digitalis. |
|---|---|
| 1st. Type unalterable; to this all digitaline may be reduced. | 1st. No standard of comparison. |
| 2nd. Constant action. | 2nd. Uncertain action; depends on the quality of the plant. |

| | |
|---|---|
| 3rd. Possibility of determining comparative excellence by the bitterness. | 3rd. No mode of determining the quality of different specimens. |
| 4th. Agreeable form. | 4th. Disagreeable smell and odor. |

M. Bouillaud states that during four or five years, not a day has passed without his employing digitaline on many patients with diseases of the heart or vessels. He has given it to from 150 to 200 patients of all ages. In all excepting three the pulse was reduced. Two of these had endocarditis and pericarditis. If the pulse was irregular previous to the taking of the digitaline, it become regular as the medicine took effect. In fifteen cases taken at hazard, in La Charite, the maximum pulse before the action of the digitaline was 96, after the medicine 41 pulsations less. In three cases the pulse was reduced 80, 102, and 106 beats. The minimum reduction in three other cases was 12, 14, 16. The number of the granules taken daily was from 2 to 7; the number of days on which the granules were taken usually 13 to 14. One patient took 70 granules in 18 days. Another 82 in 14 days. A third 98 granules in 20 days. A fourth 164 granules in 40 days without harm. As soon as pain in the head, vertigo, or nausea came on, the medicine was stopped.

M. Andral, also, in the *Union Medicela*, No. 52, 1851, gives his experience of the action of the granules on 19 patients, with either heart disease, albumenuria, phthisis, pleurisy or acute rheumatism. Two granules sometimes caused sickness. Usully three or four granules were taken. One patient took twelve daily. The greatest number of granules taken by different patients were 23, 33, 44, 50. 80. The action on the pulse was perceptible in the decrease of the number of beats from day to day. The following table of the reduction of the pulse in different cases is given:—

| Nature of Disease. | Minimum of pulse before treatm't. | Maximum of pulse during treatm't. | Difference. |
|---|---|---|---|
| Disease of heart - - | 108 | 68 | 40 |
| " - - - - - | 92 | 72 | 20 |
| " - - - - - | 80 | 68 | 12 |
| " - - - - - | 69 | 51 | 25 |
| " - - - - - | 104 | 100 | 4 |
| " - - - - - | 64 | 60 | 4 |
| " - - - - - | 44 | 44 | 0 |
| " - - - - - | 76 | 58 | 18 |
| Phthisis - - - - - | 84 | 76 | 8 |
| " - - - - - | 68 | 64 | 4 |
| Pleurisy - - - - - | 108 | 100 | 8 |
| " - - - - - | 108 | 116 | 8 |

| | | | |
|---|---|---|---|
| Hydatid of Pleura - - | 100 | 96 | 4 |
| Rheumatism of one joint | 96 | 80 | 16 |
| " in several joints - | 96 | 80 | 16 |
| Anæmia - - - - - | 80 | 76 | 4 |

The granules increase the freqency of making water from 4 to 5 times daily to 12 or 14 times. They do not always increase the quantity of urine, but sometimes they increase the quantity to two, three, or even four times the amount previously passed.

The granules act on the nervous system in different patients very differently. Sometimes, in some persons causing sleepiness and heaviness; in others, pain in the head, uneasiness, and loss of sleep.

The following account of the poisoning from taking an excess of these granules is given by Dr. Leroux in the *Union Medicale*, No. 99, p. 398.

A strong man, æt. 72, suffered from old pleurisy of the left side with œdema of the feet; his pulse was from 70 to 68. At 6 a. m., on the 25th of May he took many granules of digitaline; at 10 a. m. he took as many more as made up 30; at midday poisoning symptoms began; at 5 p. m. a physician was called, then there was pain in the head, imperfect sight, extreme præordial pain with extreme restlessness. At 9 p. m. the tongue was red and dry, there was dislike of any kind of drink, sickness, pulse 45 to 50; excessive acute pain in the head; giddiness when the patient sat up so that he had to lie down immediately. There was general weakness with drowsiness and scanty urine. The following day the pulse was 54, the general symptoms were less severe. It was the 1st of June bfoere he entirely recovered.

From these experiments and observations on the action of the granules of digitaline, it is evident that the active principle of the digitalis may be insulated and employed as a remedy, and as morphine and quinine are of more definite composition and therefore of more certain action than opium and bark, so in nearly the same degree is digitaline more definite and certain in its action than the digitalis from which it may be extracted.

I may conclude this subject by bringing to your notice some very interesting observations of Dr. Traube, of Berlin, on the reduction of the temperature of the body by the action of digitalis; the determination of the animal heat was always made in the axilla, and in twelve cases of acute rheumatism, the rate of the pulse and the temperature of the body were taken with extreme accuracy, morning and evening; half a drachm of the leaves of digitalis were infused in four ounces of water, and every two hours half an ounce of the infusion was given. The result was, that generally the temperature fell at the same time, or shortly after the digitalis produced this effect on the heart. Hence, without doubt, the reduction of the temperature was a consequence of the slower current of the blood which was produced by the action of the digitalis on the regulatory system of nerves of the heart.

Careful observation showed that digitalis caused a reduction of the temperature in the most different kinds of febrile complaints. Even in puerperal fever the temperature falls when the digitalis is in action.

Dr. Traube draws the following comparison between blood-letting and digitalis as antiphlogistics. Bleeding, while it lessens the force of the pulse, reduces the specific gravity of the blood and in low inflammations increases the tendency to serous effusion. Hence, in all low inflammations, digitalis is to be preferred as an antiphlogistic to bleeding. Moreover, the effect of venesection is much more rapid, and much more transitory; in its antiphlogistic action digitalis bears a close resemblance to antimony; but it is far less likely to effect the bowels, and hence, in all inflammatory diseases complicated with any affection of the bowels, digitalis is to be preferred to antimony. The employment of the digitalis is, however, accompanied by its own inconveniences and even dangers. It may produce sudden prostration of nervous and muscular action, and even syncope and death; that is, the stimulating action on the regulatory system of nerves may suddenly give place to the paralyzing action. Hence arises the necessity for watching those who are taking this medicine; they should be seen at least twice daily, and if there be sickness, or irregularity in the rhythm of the heart, or great reduction in the rate of the pulse, the medicine must be omitted.—*Virgina Med. Jour.*

